# Soluble urokinase plasminogen activator receptor in vaginally collected amniotic fluid predicting fetal inflammatory response syndrome: a prospective cohort study

**DOI:** 10.1186/s12884-023-06221-0

**Published:** 2024-01-10

**Authors:** Violeta Gulbiniene, Irena Dumalakiene, Greta Balciuniene, Ingrida Pilypiene, Ieva Narkeviciute, Vitalij Novickij, Gintautas Vysniauskis, Diana Ramasauskaite

**Affiliations:** 1 Faculty of Medicine, Vilnius University, Vilnius, Lithuania; 2 Department of Immunology, State Research Institute Center of Innovative Medicine, Vilnius, Lithuania

**Keywords:** Amniotic fluid, Biomarker, FIRS, IL-6, MMP-8, Preterm premature rupture of membranes, suPAR, TNF-α

## Abstract

**Background:**

Improving noninvasive antenatal diagnosis of fetal inflammatory response syndrome (FIRS) can assist in the evaluation of prenatal risk and reduce perinatal outcomes. This study aimed to determine whether soluble urokinase-type plasminogen activator receptor (suPAR) in vaginally collected amniotic fluid is significant in identifying FIRS after preterm premature rupture of membranes before 34 weeks of gestation.

**Methods:**

This was a prospective cohort study of 114 pregnant women and their newborns after preterm premature rupture of membranes at 22–34^+6^ weeks of gestation. SuPAR was evaluated using an enzyme-linked immunosorbent assay in vaginally collected amniotic fluid. Patients were classified according to the presence or absence of FIRS. FIRS was defined by umbilical cord blood interleukin-6 level > 11 pg/mL or histological funisitis. The data were analyzed using the R package (R–4.0.5).

**Results:**

SuPAR was detected in all amniotic fluid samples with a median of 26.23 ng/mL (interquartile range (IQR), 15.19–51.14). The median level of suPAR was higher in the FIRS group than in the non-FIRS group, 32.36 ng/mL (IQR, 17.27–84.16) vs. 20.46 ng/mL (IQR, 11.49–36.63) (*P* = 0.01), respectively. The presence of histological chorioamnionitis significantly increased the suPAR concentration in the FIRS group (*P* < 0.001). The areas under the curve for FIRS and FIRS with histological chorioamnionitis were 0.65 and 0.74, respectively, with an optimum cutoff value of 27.60 ng/mL. Controlling for gestational age, the cutoff of suPAR more than 27.60 ng/mL predicted threefold higher odds for FIRS and sixfold higher odds for FIRS with histologic chorioamnionitis.

**Conclusion:**

Soluble urokinase-type plasminogen activator receptor in vaginally obtained amniotic fluid may assist in evaluating prenatal risk of FIRS in patients after preterm premature rupture of membranes before 34 weeks of gestation.

## Background

Fetal inflammatory response syndrome (FIRS) represents a systemic fetal response to intra-amniotic infection and/or inflammation. It is associated with preterm premature rupture of membranes (PPROM) and preterm delivery and increases the risk of adverse neonatal outcomes [1, 2]. FIRS is defined as an elevation of cytokines in fetal blood [3] or identified histologically as funisitis [4]. In response to intraamniotic infection, the production of proinflammatory cytokines (interleukin-6 (IL-6), tumor necrosis factor-α (TNF-α), etc.) contributes to preterm delivery by stimulating prostaglandin synthesis and myometrial contractions and inducing the release of matrix metalloproteinases, which cause membrane rupture and cervical ripening [5]. This leads to an increase in inflammatory cells, cytokines, or enzymes in the amniotic fluid [6]. There is mounting evidence indicating that the analysis of inflammatory biomarkers in amniotic fluid appears to be the optimal approach to diagnosing intraamniotic infection and/or inflammation and FIRS [1, 3, 7–10].

The plasminogen activator system, consisting of urokinase-like plasminogen activator (uPA), its receptor (uPAR), and an inhibitor, participates in the processes of cell adhesion, migration, and invasion, which are essential for inflammation [11]. In a membrane-bound form, uPAR is expressed on immune cells (activated T cells, neutrophils, macrophages, etc.) and other cells (trophoblast cells, fetal membrane cells, etc.) [11–13]. Cleaved uPAR becomes soluble urokinase plasminogen activator receptor (suPAR) and is found in various body fluids, including blood, urine, saliva, and cerebrospinal fluid [12, 14, 15]. Numerous studies have demonstrated that suPAR is a nonspecific marker of immune activation and systemic inflammation in various infectious, autoimmune, and malignant diseases [12, 16, 17]. In addition, uPA/uPAR activity in local fibrinolysis and proteolysis contributes to processes of reproduction: ovulation, implantation and placentation, tissue remodeling, and angiogenesis [13]. The plasminogen activator system is thought to be involved in the development of pregnancy and its inflammatory complications (preeclampsia) [13, 18, 19]. However, to the best of our knowledge, no research has been done to assess the association of amniotic fluid suPAR and FIRS.

In this study, we aimed to evaluate the levels of suPAR in vaginally collected amniotic fluid and whether suPAR is of value in identifying FIRS after PPROM. Furthermore, we sought to compare the association of suPAR with other amniotic inflammatory biomarkers, such as IL-6, TNF-α, and matrix metalloproteinase-8 (MMP-8). The significance of these biomarkers in noninvasively obtained amniotic fluid predicting FIRS was reported previously [20, 21].

## Materials and methods

**Fig. 1:**
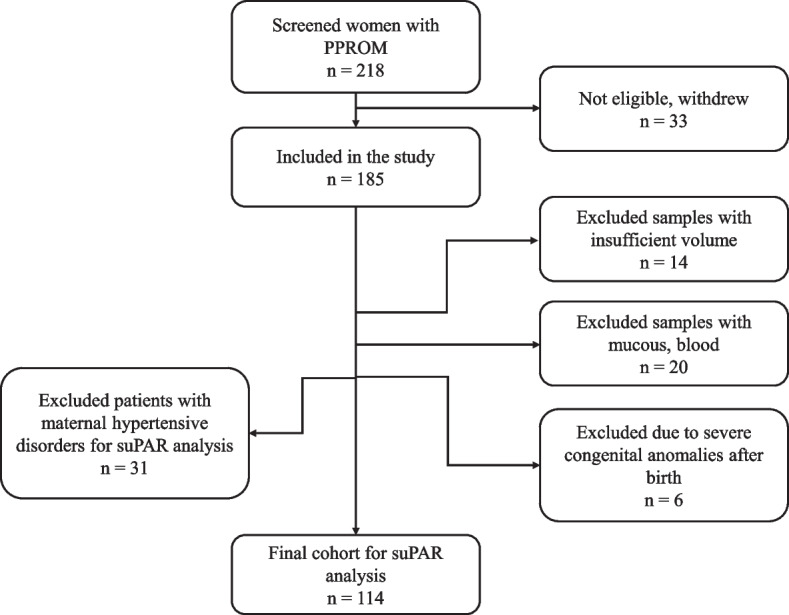
The patient flow diagram

The study population consisted of singleton pregnant women with PPROM at 22–34^+6^ weeks of gestation who were admitted to Vilnius University Hospital Santaros Klinikos between 2017 and 2020 and their newborns after delivery. The exclusion criteria were multiple gestations, vaginal bleeding, placenta previa, fetal and neonatal malformations, non-reassuring fetal status. Patients with inadequate amniotic fluid samples, such as insufficient volume or containing mucous and/or blood, were also excluded. Additional amniotic fluid biomarkers, whose prognostic value has already been reported in previous publications [20, 21], were evaluated during the study. For the assessment of suPAR, due to the association of suPAR with hypertensive diseases, we retrospectively excluded all cases with maternal hypertensive disorders for the suPAR analysis [19]. Figure 1 shows the patient flow diagram with a total of 114 participants in the final analysis. The study was approved by the Vilnius Regional Biomedical Research Ethics Committee (2017–07-04 No. 158200–17-931–434). All participants provided informed written consent before enrollment.

Based on the last menstrual cycle, gestational age (GA) was calculated and confirmed or adjusted by an ultrasound scan between 11^+0^ and 13^+6^ weeks of pregnancy. Premature rupture of membranes was determined with a sterile speculum confirming amniotic fluid pooling in the vagina. In uncertain cases, the presence of placental alpha microglobulin-1 protein (Amnisure, QIAGEN, Germantown, MD, USA) in vaginal fluid confirmed the rupture of membranes. Women with PPROM before 34 weeks of gestation were on expectant management and received antibiotics, one course of prenatal corticosteroids, and, if necessary, tocolytics during the lung maturation course, in accordance with the hospital's protocol. Antibiotic therapy included intravenous ampicillin (2 g every 6 h) and erythromycin (250 mg every 6 h) for 48 h followed by oral amoxicillin (500 mg every 8 h) and erythromycin (250 mg every 6 h) for five days. Dexamethasone was injected intramuscularly in two 12-mg doses every 12 h for fetal lung maturation. After fetal lung maturation, labor started spontaneously or was induced. The indications for labor induction were intrauterine infection according to Gibb's criteria, bleeding, or non-reassuring fetal status. There were no changes in routine clinical practice following participation in the study.

Free leaking amniotic fluid was collected vaginally with a sterile speculum into the centrifuge tube every 2 days. The last sample obtained within 48 h before labor was included in further analysis. We chose a sampling period of less than 48 h to maintain a significant timing relationship between the results of amniotic fluid tests and histological findings of the placenta and umbilical cord at birth. To minimize contamination and attain clear specimens, samples were centrifuged at 3000 rpm for 5 min at 4 °C and stored at − 80 °C.

Immunological assays of stored amniotic fluid samples were performed at the State Research Institute, Centre for Innovative Medicine. The levels of biomarkers were measured using an enzyme-linked immunosorbent assay (ELISA) with a commercial kit (suPAR: R&D Systems, Minneapolis, MN, USA; IL-6, TNF-α and MMP-8: Bender MedSystems, Vienna, Austria). Nondiluted specimens were used for TNF-α and IL-6 ELISA assays. Samples for suPAR analysis were diluted to 1:5, and specimens for MMP-8 analysis were diluted to 1:10. If the measured concentrations of analytes exceeded the highest point on the standard curve, dilutions of 1:2, 1:5, 1:10 or 1:100 were performed. Diluents were provided by the manufacturer. The concentrations of markers were calculated according to standard curves by a special program for the evaluation of ELISA results: Gen5 Microplate Data Collection & Analysis Software (BioTek Instruments, Winooski, VT, USA). All samples were assayed in duplicate. The intra- and interassay coefficients of variation were each < 10%.

FIRS was defined according to umbilical cord blood IL-6 levels > 11 pg/mL and/or histological funisitis [3, 4, 22]. After birth, the IL-6 concentration was determined in umbilical cord serum by automated chemiluminescent enzyme immunoassay using a kit (DPC, Los Angeles, CA, USA). A histological examination of the placenta and umbilical cord was performed. Funisitis was identified by neutrophilic infiltration in the umbilical vascular wall or Wharton's jell. Histological chorioamnionitis was defined by the infiltration of neutrophils into the choriodecidua and amnion. Neonates were evaluated and followed up from birth until discharge from the hospital. The concentrations of biomarkers were concealed from researchers and clinical personnel.

Statistical analysis was performed using R software version R–4.0.5 (R Core Team, 2021). Baseline differences between groups were determined using Student’s t, Mann–Whitney–Wilcoxon, Kruskal–Wallis, χ2, or Fisher’s exact tests as appropriate. We used receiver operating characteristic (ROC) curve analysis to evaluate the ability of variables to discriminate between groups and DeLong test to compare the area under the curve (AUC) of different models. The Youden index determined the best cutoff values. Using logistic regression analysis, we estimated odds ratio (aOR) with 95% confidence interval (CI) of FIRS and histologic chorioamnionitis and compared these models with ANOVA. Random Forest analysis was conducted to predict FIRS and to rank the importance of predictors. A SHAP summary plot was generated to visualize the impact of individual features on the Random Forest model's predictions. The x-axis demonstrates Shapley values (impact on the model output and direction). On the y-axis, the features (predictors) are listed in descending order of their importance in the Random Forest model. The colors represent the feature values of predictors: higher values are in yellow, and lower values are in dark purple. Spearman correlation analysis was used to identify the correlation between immunological markers and other parameters *P* < 0.05 was considered statistically significant.

## Results

The study cohort included 114 women with PPROM before 34^+6 days^ weeks of gestation and their newborns: the FIRS group (*n* = 48) and the non-FIRS group (*n* = 66). Table 1 displays the clinical characteristics of the study population.

**Table 1:** Demographic and clinical characteristics of the study population

Characteristics	FIRS group (*n* = 48)		Non-FIRS group (*n* = 66)		*P* value
Maternal characteristics					
Maternal age, years, mean ± SD	31.2	± 5.5	31.1	± 5.7	0.93
Latency period^a^, hours, median, IQR	23	4–51	26	11–59	0.12
Gestational diabetes, n, %	10	21	13	20	0.91
Group B streptococcus test positive, n, %	7	24	10	22	0.81
Primigravida, n, %	17	46	31	40	0.57
Primiparous, n, %	20	39	28	44	0.57
Gestational age at PPROM, weeks, median, IQR	31	27–33	33	32–34	0.001
Tocolytics, n, %	23	48	38	58	0.31
Administration of antibiotics, n, %	48	100	66	100	0.45
Antenatal glucocorticosteroids, n, %	41	85	55	83	0.76
Mode of delivery, n, %					
vaginal delivery	43	90	55	83	0.42
cesarean section	5	10	11	17	
Induction of delivery or cesarean section n, %	17	35	33	50	0.28
Spontaneous delivery n, %	31	65	33	50	
Cause of initiating delivery, n, %					
Elevating maternal blood biomarkers^b^	10	58	9	27	0.13
≥ 34 weeks of gestation	2	12	7	21	
Prolonged latency period (> 7 days)	2	12	3	9	
Maternal condition	1	6	10	30	
Worsening fetal condition	2	12	3	9	
Umbilical cord prolapse	0	0	1	3	
Clinical chorioamnionitis, n, %	6	13	1	2	0.02
Histological chorioamnionitis, n, %	38	79	15	23	< 0.001
Funisitis, n, %	21	44	0	-	< 0.001
Neonatal characteristics					
Gestational age at birth, weeks, median, IQR	31	27–33	33	32–34	0.001
Birthweight, grams, mean ± SD	1688	± 669	2070	± 555	0.01
Apgar scores < 7 at 1 min., n, %	14	29	6	9	0.01
Apgar scores < 7 at 5 min., n, %	6	13	1	2	0.04
Umbilical cord arterial pH, Median, IQR	7.35	7.29–7.42	7.36	7.32–7.40	0.77
Major outcome^c^, n, %	19	40	11	17	0.01
Respiratory distress, n, %	46	96	48	73	0.001
Severe respiratory distress syndrome^d^, n, %	10	21	8	12	0.01
Respiratory support, n, %					
None	2	4	18	27	0.01
Mechanical ventilation	11	23	6	9	
Noninvasive respiratory therapy	35	73	42	64	
Neonatal death, n, %	1	2	0	-	0.42
Sepsis, n %	8	17	2	3	0.02
Early-onset sepsis	7	17	1	2	0.01
Bronchopulmonary dysplasia, n, %	9	19	4	6	0.04
Open ductus arteriosus, n %	8	17	8	12	0.46
Retinopathy of prematurity	15	32	6	9	0.01
Intraventricular hemorrhage, n, %					
None	29	60	46	70	0.24
1–2 grade	14	29	18	27	
3–4 grade	5	10	2	3	

*FIRS* fetal inflammatory response syndrome, *SD* standard deviation, *IQR* interquartile range

^a^The time from preterm premature rupture of the membrane to delivery

^b^C-reactive protein, white blood cell count, and neutrophil count

^c^Major neonatal outcomes were diagnosed if one or more of the following occurred: severe respiratory distress syndrome, the need for mechanical ventilation, death, early sepsis, early hypotension, severe intraventricular hemorrhage, bronchopulmonary dysplasia, and severe retinopathy of prematurity

^d^The criteria for severe respiratory distress syndrome (Grade 3 and 4) included opaque lungs and/or alveolar shadowing obscuring the cardiac border in the chest radiogram

There were no differences in most maternal factors between the groups, except for histological chorioamnionitis, which was more common in the FIRS group. Newborns in the FIRS group had lower birth weight and gestational age and were likely to have lower Apgar scores than newborns without FIRS. The incidence of respiratory problems, especially severe RDS, early-onset sepsis, bronchopulmonary dysplasia, and retinopathy of prematurity, was higher in the FIRS group than in the non-FIRS group. The median umbilical cord arterial pH and rates of neonatal death, patent ductus arteriosus, and intraventricular hemorrhage did not differ between the groups.

**Fig. 2:**
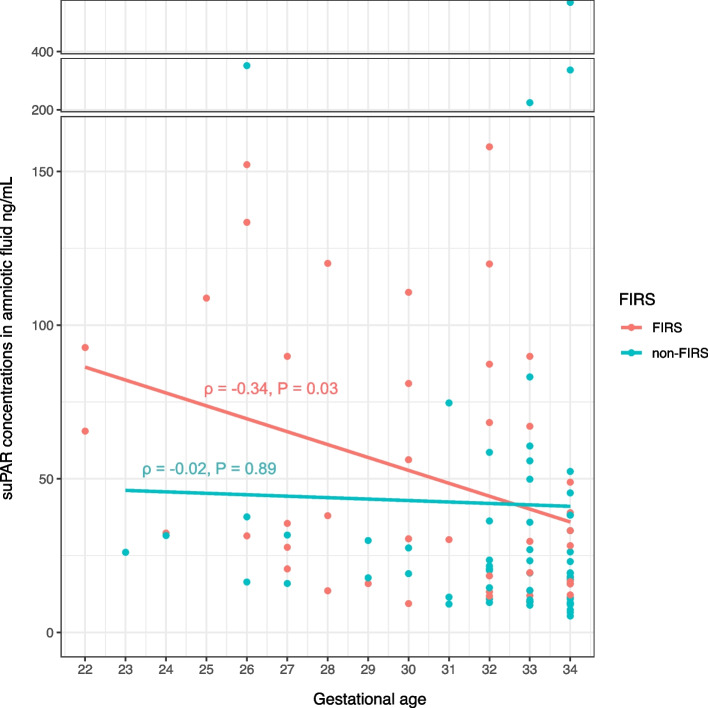
The relationship between suPAR levels and gestational age according to FIRS. The scatter plot with the regression line in red depicts the correlation between suPAR and gestational age in the FIRS group (ρ = -0.34, *P* = 0.03). The scatter plot with the regression line in blue illustrates the correlation between suPAR and gestational age in the non-FIRS group (ρ = -0.02, *P* = 0.89). suPAR, soluble urokinase plasminogen activator receptor; FIRS, fetal inflammatory response syndrome

We detected suPAR in all amniotic fluid samples in the 5.36–399.98 ng/mL range with a median of 26.23 ng/mL (interquartile range (IQR), 15.19–51.14). There was a weak negative correlation between suPAR levels and gestational age (ρ = -0.29, P = 0.01). Higher levels of suPAR were associated with lower gestational age (median of 33.93 ng/mL for 22–27 weeks of GA, median of 29.94 ng/mL for 28–31 weeks of GA and median of 19.83 ng/mL for 32–34 weeks of GA, (Kruskal, *P* = 0.02)). Considering the possible effect of inflammation on this association, we assessed the correlation between suPAR and gestational age by FIRS groups: a weak negative correlation between suPAR and gestational age remained in the FIRS group (ρ = -0.34, *P* = 0.03), whereas there was no association between parameters in the non-FIRS group (ρ = -0.02, *P* = 0.89) (Fig. 2).

**Fig. 3:**
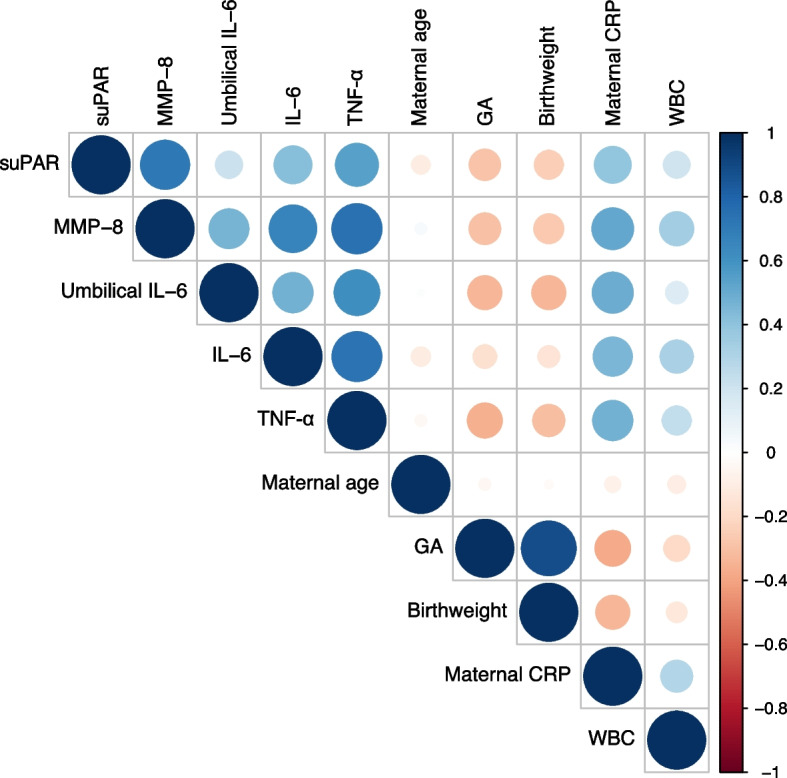
The correlation matrix of suPAR levels with other inflammatory biomarkers and maternal and neonatal parameters. In the matrix, each circle indicates the correlation between two variables. Blue represents positive correlations, and red signifies negative correlations, with color intensity and circle size reflecting the strength of correlation coefficients. The legend on the right displays correlation coefficients alongside their respective colors. The correlation coefficients and P values among variables are as follows: suPAR and MMP-8 (ρ = 0.71, *P* < 0.001); suPAR and umbilical IL-6 (ρ = 0.22, *P* = 0.05); suPAR and amniotic fluid IL-6 (ρ = 0.42, *P* < 0.001); suPAR and TNF-α (ρ = 0.54, *P* < 0.001); suPAR and maternal age (ρ = -0.10, *P* = 0.31); suPAR and maternal C-reactive protein (ρ = 0.39, *P* < 0.001); suPAR and maternal white blood cell count (ρ = 0.21, *P* = 0.04); suPAR and gestational age (ρ = -0.29, *P* = 0.01); suPAR and birthweight (ρ = -0.25, *P* = 0.01). suPAR, soluble urokinase plasminogen activator receptor; MMP-8, matrix metalloproteinase-8; IL-6, interleukin-6; TNF-α, tumor necrosis factor-α; CRP, C-reactive protein; WBC, white blood cells

Spearman correlation analysis was conducted to assess the association of suPAR with other biomarkers, as well as maternal and neonatal parameters, as depicted in Fig. 3. The figure illustrates a correlation matrix, a tabular representation showing correlation coefficients among variables. In the matrix, each circle indicates the correlation between two variables. Blue represents positive correlations, and red signifies negative correlations, with color intensity and circle size reflecting the strength of correlation coefficients. The legend on the right displays correlation coefficients alongside their respective colors. There was a strong positive suPAR correlation with MMP-8; a positive moderate correlation with vaginal amniotic fluid TNF-α, IL-6, and maternal C-reactive protein; a positive weak correlation with maternal leukocyte count, and a negative weak correlation with gestational age and neonatal birthweight.

**Fig. 4:**
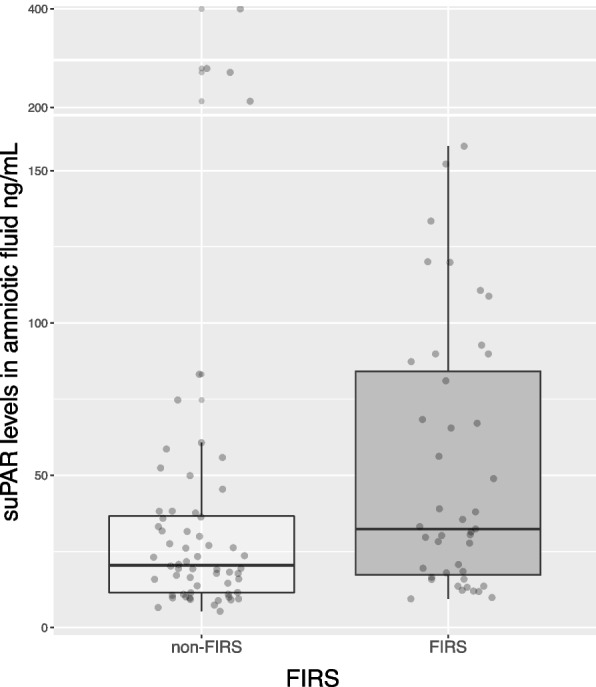
Amniotic fluid suPAR levels in fetal inflammatory response syndrome. The median concentration of suPAR in the FIRS group and the non-FIRS group: median, 32.36 ng/mL, IQR, 17.27–84.16, vs. median, 20.46 ng/mL, IQR, 11.49–36.63, respectively (Wilcoxon, *P* = 0.01). suPAR, soluble urokinase plasminogen activator receptor; FIRS, fetal inflammatory response syndrome; IQR, interquartile range

**Fig. 5:**
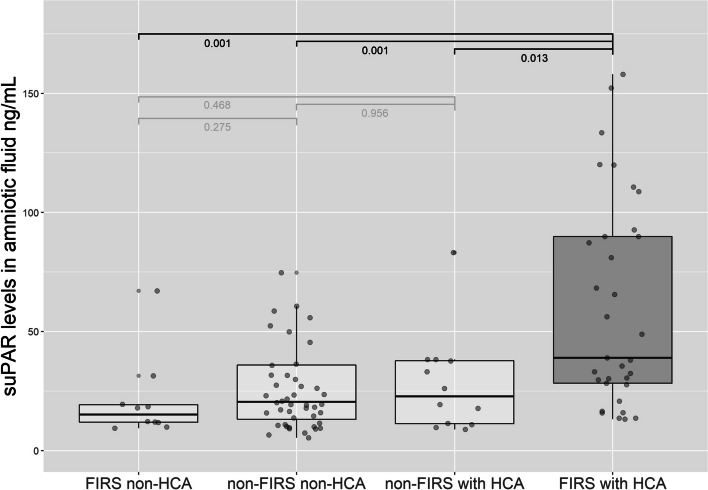
SuPAR concentrations in FIRS groups according to the presence or absence of histological chorioamnionitis: FIRS with histological chorioamnionitis, median, 38.98 ng/mL, IQR, 28.25–89.85, vs. FIRS without histological chorioamnionitis, median, 15.10, IQR, 11.87–19.21, vs. non-FIRS with histological chorioamnionitis, median, 22.73 ng/mL, IQR, 11.30–37.77, vs. non-FIRS no histological chorioamnionitis, median, 20.46 ng/mL, IQR, 13.17–35.97; Kruskal, *P* < 0.001. suPAR, soluble urokinase plasminogen activator receptor; FIRS, fetal inflammatory response syndrome; IQR, interquartile range.; HCA, histological chorioamnionitis; IQR, interquartile range

Figure 4 illustrates suPAR concentrations in vaginally collected amniotic fluid according to the presence or absence of FIRS. The median level of suPAR was significantly higher in patients with FIRS than in the non-FIRS group, 32.36 ng/mL (IQR, 17.27–84.16) vs. 20.46 ng/mL (IQR, 11.49–36.63) (Wilcoxon, *P* = 0.01), respectively. Moreover, the presence of histological chorioamnionitis was associated with a significant increase in suPAR levels in the FIRS group. Figure 5 shows suPAR levels in vaginally collected amniotic fluid according to the presence or absence of FIRS and histologic chorioamnionitis. Patients with FIRS and histologic chorioamnionitis were more likely to have significantly higher suPAR concentrations in amniotic fluid (Kruskal, *P* < 0.001). There were no statistically significant differences in suPAR levels between participants with FIRS but without histologic chorioamnionitis or without FIRS but with histologic chorioamnionitis or without these outcomes.

ROC curves were constructed to select the cutoff value predicting FIRS, as well as FIRS with concomitant histological chorioamnionitis (AUC for FIRS: 0.65, 95% CI 0.54–0.76; AUC for FIRS with histological chorioamnionitis: 0.74, 95% CI 0.64–0.84). Table 2 provides the suPAR cutoff value > 27.60 ng/ml and its diagnostic parameters for FIRS and concomitant histological chorioamnionitis. Diagnostic suPAR parameters detecting FIRS with histological chorioamnionitis together were better than identifying FIRS alone. However, there was no significant difference between these AUCs with the DeLong test (*P* = 0.20).

**Table 2:** The cutoff values and diagnostic characteristics of inflammatory markers identifying FIRS and histological chorioamnionitis

Cutoff value		AUC		Sensitivity		Specificity		PPV		NPV		Delong test *P* value
				%	95% CI	%	95% CI	%	95% CI	%	95% CI	
FIRS												
suPAR	27.60	0.65	0.54–0.76	65	51–79	65	53–77	57	48–68	72	64–82	0.20^a^
MMP-8	190.91	0.76	0.66–0.86	82	68–93	67	55–79	65	57–75	34	73–93	0.05^b^
TNF-α	86.95	0.82	0.72–0.91	75	61–86	86	78–95	81	70–91	82	75–90	0.01^b^
IL-6	27843.18	0.77	0.68–0.86	50	35–65	95	89–100	89	77–100	72	67–79	0.05^b^
FIRS with histological chorioamnionitis												
suPAR	27.60	0.74	0.64–0.84	79	64–91	67	56–79	53	44–64	88	80–94	0.20^a^
MMP-8	171.18	0.84	0.75–0.92	94	85–100	59	47–71	53	46–61	95	88–100	0.14^c^
TNF-α	97.89	0.84	0.76–0.92	76	62–91	53	46–61	71	59–83	88	81–95	0.11^c^
IL-6	2695.68	0.82	0.73–0.91	61	45–76	95	88–100	85	72–96	83	77–89	0.30^c^

*MMP-8* matrix metalloproteinase-8, *TNF-α* tumor necrosis factor-α, *IL-6* interleukin-6, *FIRS* fetal inflammatory response syndrome, *AUC* area under the curve, *CI* confidence intervals, *PPV* positive predictive value, *NPV* negative predictive value

^a^*P* value represents the Delong test between suPAR ROC curve for FIRS compared to suPAR ROC curve for FIRS with histological chorioamnionitis

^b^*P* value represents the suPAR ROC curve for FIRS compared to the ROC curve for FIRS of the other biomarkers

^c^*P* value represents the suPAR ROC curve for FIRS with histological chorioamnionitis compared to the ROC curve for FIRS with histological chorioamnionitis of the other biomarkers

We compared the ability of suPAR to identify FIRS and FIRS with histological chorioamnionitis with that of other inflammatory biomarkers, such as MMP-8, TNF-α, and IL-6 (Table 2). The Delong test for two correlated ROC curves was performed between suPAR ROC curves for FIRS and FIRS with histological chorioamnionitis and ROC curves of other biomarkers. All three biomarkers had better diagnostic parameters than suPAR for identifying FIRS. In contrast, the suPAR AUC was not significantly different from that of MMP-8 and IL-6 for identifying FIRS with histological chorioamnionitis.

Using a suPAR cutoff value of > 27.60 ng/mL and controlling for gestational age, we constructed logistic regression models for FIRS and FIRS with histological chorioamnionitis. Logistic regression analysis revealed that elevated suPAR concentrations increased the odds of having FIRS, as well as FIRS with histological chorioamnionitis (Table 3). Controlling for GA, a suPAR level of > 27.60 ng/mL increased the odds of FIRS 2.8 times, whereas the odds for FIRS with histological chorioamnionitis increased 6.0 times. In both predictive models, gestational age had negative estimated coefficients; the higher the gestational age was, the lower the odds of outcomes were. Overall, in the logistic regression, the amniotic fluid suPAR cutoff concentration of more than 27.60 ng/mL significantly predicted the odds for FIRS and FIRS with concomitant histological chorioamnionitis, controlling for GA.

**Table 3:** Logistic regression for FIRS and histological chorioamnionitis with suPAR cutoff > 27.60 ng/mL, controlling for gestational age

Models	Coefficients	Estimate	Standard errors	Z value	P value	aOR	95% CI
FIRS	suPAR > 27.60	1.01759	0.44	2.33	0.02	2.77	1.18– 6.61
	GA	-0.14846	0.07	-2.07	0.04	0.86	0.74–0.99
FIRS with histological chorioamnionitis	suPAR > 27.60	1.79240	0.51	3.50	< 0.001	6.00	2.28–17.36
	GA	-0.17678	0.08	-2.33	0.02	0.84	0.72–0.97

*aOR* adjusted odds ratio, *CI* confidence interval, *GA* gestational age, *FIRS* fetal inflammatory response syndrome

**Fig. 6:**
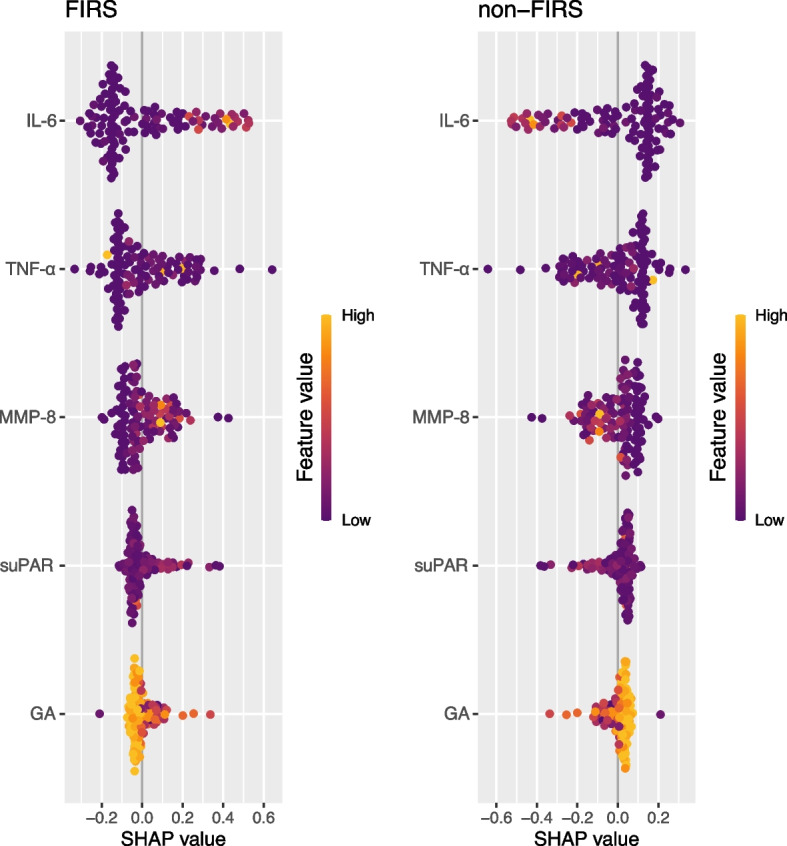
SHAP Summary Plot for Variable Importance. The x-axis demonstrates Shapley values (impact on the model output and direction). Positive values contribute to increasing the chances of FIRS, while negative values contribute to decreasing it. On the y-axis, the predictors (IL-6, TNF-α, suPAR, MMP-8, and gestational age) are listed in descending order of their importance in the Random Forest model. The colors represent the feature values of predictors: higher values are in yellow, and lower values are in dark purple. suPAR, soluble urokinase plasminogen activator receptor; MMP-8, matrix metalloproteinase-8; IL-6, interleukin-6; TNF-α, tumor necrosis factor-α; GA, gestational age; FIRS, fetal inflammatory response syndrome

Based on our previous findings, we performed Random Forest analysis to predict FIRS and to rank the importance of variables using five predictors, as follows: suPAR, IL-6, TNF-α, MMP-8, and GA. We tested a model with 500, 1000, 2000, and 3000 trees, and the number of variables to be tested (mtry) was set as the square root of the total number of predictors (the square root of 5 equals 2) with nearby values from 1 to 5. We verified the model using the Out-of-Bag error (OOB). The OOB error rate was lowest when mtry was 2, and it did not improve building model with an increasing number of trees. A SHAP summary plot was generated to visualize the impact of individual features on the model's predictions (Fig. 6). The x-axis demonstrates Shapley values (impact on the model output and direction). Figure 6 demonstrates that out of the 5 variables selected, higher values of IL-6 and TNF-α were found to be the most important variables in predicting FIRS. Meanwhile, suPAR and MMP-8 performed moderately. GA was the least important predictor of FIRS with negative direction; FIRS was less expected with higher GA. In summary, Random Forest analysis revealed that suPAR, in vaginal amniotic fluid, holds moderate significance in predicting FIRS compared to the other analyzed biomarkers.

## Discussion

In this PPROM cohort, suPAR was detected in all noninvasively obtained vaginal amniotic fluid samples. The results revealed an association between elevated vaginal amniotic fluid suPAR concentrations and FIRS, as well as FIRS with histological chorioamnionitis. Particularly, controlling for GA, vaginal amniotic fluid suPAR concentration of more than 27.60 ng/mL predicted approximately threefold greater odds of having FIRS and sixfold greater odds of having FIRS with histological chorioamnionitis after PPROM before 34 weeks of gestation. In addition, we observed a strong to moderate positive correlation of suPAR with other inflammatory biomarkers (MMP-8, TNF-α) in vaginal amniotic fluid.

Although previous studies determined suPAR concentrations in various body fluids [12, 14, 15], suPAR levels in vaginal amniotic fluid have not been investigated and quantified before. In this study, we found suPAR in all vaginal amniotic fluid samples after preterm rupture of membranes. The median suPAR level of 26.23 ng/mL (IQR, 15.19–51.14) was higher than that in previous studies that reported blood levels of 2.02–4.4 ng/mL in pregnant women [18, 19]. Our results correspond with Uszynski’s research, which determined 100–200 times higher concentrations of uPA and uPAR in gestational tissues and amniotic fluid obtained during cesarean section than in plasma. The authors found uPAR in all amniotic fluid samples at a level of 2.78 ± 1.06 ng/mg of protein [13]. Higher suPAR levels in amniotic fluid might be due to uPAR expression on trophoblasts and fetal membrane cells, as well as due to known uPA/uPAR activity in fibrinolytic processes present in PPROM. Collectively, these data suggest that suPAR is found at relatively high levels in the vaginal amniotic fluid after membrane rupture.

Furthermore, in vaginal amniotic fluid, suPAR levels correlated with other inflammatory markers, such as MMP-8, TNF-α, and IL-6. These findings are consistent with observations in other inflammatory conditions where blood suPAR levels were associated with levels of proven inflammatory markers, such as TNF-α, leukocyte count, and C-reactive protein [19, 23–25]. The strongest correlation between suPAR and MMP-8 may be explained by the fact that both systems (plasminogen activation system and matrix metalloproteinases) are involved in the degradation of the extracellular matrix and plasmin participates in metalloproteinase activation [11]. Moreover, there is a lack of information on the relationship of suPAR with gestational age. In our study, there was a weak negative correlation between suPAR and gestational age. Odden et al. reported that suPAR tended to be inversely associated with the duration of gestation, although it was not statistically significant [18]. We determined a weak inverse correlation between suPAR and gestational age in the FIRS group, whereas there was no association in the non-FIRS group. Overall, suPAR levels demonstrated a somewhat scattered distribution in relation to gestational age, and the ρ coefficient of -0.34 is not sufficiently significant to draw any clinical implications.

Currently, suPAR is considered a nonspecific biomarker of systemic chronic inflammation, reflecting the course, severity, and prognosis of the disease [12, 25, 26]. Indeed, in our study, suPAR proved to be a significant inflammatory biomarker, indicating an increased risk of FIRS, as well as FIRS with histological chorioamnionitis. Vaginal amniotic fluid suPAR levels above 27.60 ng/ml increased the odds of having FIRS threefold and having both FIRS and histological chorioamnionitis sixfold. The highest suPAR levels were found in patients with FIRS and histological chorioamnionitis, whereas histological chorioamnionitis or FIRS alone was associated with moderately elevated suPAR. Since ascending intraamniotic infection/inflammation usually may proceed from the chorion to the fetus [6], our findings correspond with observations that suPAR is associated with more advanced disease and may assist in identifying different types and stages of inflammation [25].

Although the biomarkers of FIRS correlated strongly to moderately, each has its advantages and disadvantages. In ROC analysis, suPAR showed similarly good characteristics in diagnosing FIRS with histological chorioamnionitis, whereas MMP-8, TNF-α and IL-6 performed better in identifying FIRS alone. Random Forest analysis identified IL-6 and TNF-α as the most influential FIRS predictors among the selected variables. SuPAR and MMP-8 had moderate importance. Raggam et al. observed that suPAR better reflected the inflammatory state to IL-6, C-reactive protein, and procalcitonin [27]. According to others, suPAR has limited diagnostic value but carries superior prognostic value [25, 26]. Furthermore, most inflammatory markers are short-lived, dependent on diet, exercise, and sample handling, and their levels rise or fall as they control the acute inflammatory response. In contrast, the choice of suPAR as a biomarker is determined by its stability due to minimal circadian and day-to-day variations, concentration steadiness after repeated freezing/thawing, and a long half-time period [25, 28–30]. Although there is a significant association of vaginal amniotic fluid suPAR with FIRS and histological chorioamnionitis, signifying progressed inflammation, more studies are needed to compare its characteristics to other inflammatory biomarkers.

To the best of our knowledge, this is the first study evaluating suPAR to predict FIRS after PPROM. The strengths of the study include the noninvasive vaginal amniotic fluid analysis and the use of outcomes (FIRS and histological chorioamnionitis) defined by proven biochemical and histological criteria. The analysis of vaginal amniotic fluid has proven to be noninvasive, easily performed, informative, and without complications. Moreover, suPAR levels were blinded and did not affect patient treatment. We acknowledge the limitations of our study: the results were not confirmed by conventional methods such as amniocentesis. The strong correlation between amniocentesis and vaginal amniotic fluid biomarkers [8, 9] led us to assume that vaginal amniotic fluid reflects amniotic fluid obtained by amniocentesis. Another limitation was the relatively small sample size of only 10 cases in the FIRS with non-histological chorioamnionitis subgroup assessing suPAR levels based on the presence or absence of FIRS and histologic chorioamnionitis. This limitation restricts statistical power and generalizability. An additional constraint of study methodology lies in its applicability, specifically limited to sampling conducted within 48 h from delivery. Sampling at this timeframe resulted in the exclusion of cases with longer latency periods and low residual amniotic fluid volume. Consequently, the resulting cohort may not fully represent the population of patients diagnosed with PPROM. Future studies with a larger sample size in this subgroup would enhance the reliability of results and facilitate more comprehensive analyses.

## Conclusions

It remains challenging to determine the best approach to evaluating and treating women with PPROM. The duration of the gestation and an assessment of the risks of immediate delivery versus expectant management is the key to determining optimal management strategies [31]. Our findings indicate that suPAR is present in noninvasively obtained vaginal amniotic fluid in high concentrations in cases with FIRS and histological chorioamnionitis. Considering its molecular stability, the noninvasive analysis of vaginal amniotic fluid suPAR may assist in the evaluation of prenatal risk after PPROM. Further studies are needed to clarify whether suPAR merely indicates an increased immune response in the amniotic cavity or whether this receptor may serve as a future diagnostic or therapeutic tool.

## Data Availability

The datasets analyzed during the current study are available from the corresponding author upon reasonable request.

## Abbreviations

FIRS: Fetal inflammatory response syndrome
PPROM: Preterm premature rupture of membranes
IL-6: Interleukin-6
TNF-α: Tumor necrosis factor-α
uPA: Urokinase-like plasminogen activator
uPAR: Urokinase-like plasminogen activator receptor
MMP-8: Matrix metalloproteinase-8
GA: Gestational age
ELISA: Enzyme-linked immunosorbent assay
ROC: Receiver operating characteristic
AUC: Area under the curve
aOR: Adjusted Odds ratio
CI: Confidence interval
IQR: Interquartile range
